# Postural imbalance in patients with rheumatoid arthritis: a cross-sectional study

**DOI:** 10.1590/1806-9282.20260317

**Published:** 2026-10-05

**Authors:** Ana Luisa Trevisan, Bruna Furukawa, Clara Mayumi Mikami, Naira Alessandra Hurmus, Thelma Larocca Skare, Renato Nisihara, Barbara Stadler Kahlow

**Affiliations:** 1 Mackenzie Evangelical School of Medicine of Paraná – Curitiba (PR), Brazil.

**Keywords:** Rheumatoid arthritis, Postural balance, Accidental falls.

## Abstract

**OBJECTIVE:**

The aim of this study was to evaluate postural balance in patients with rheumatoid arthritis and identify factors associated with balance impairment.

**METHODS:**

This cross-sectional case–control study included 101 patients with rheumatoid arthritis and 94 healthy controls recruited at a single tertiary referral center between March 2024 and April 2025. Epidemiological and clinical data, including disease activity, current treatments, and the number of falls during the previous year, were obtained through medical record review and direct patient interviews. Balance was assessed using the Berg Balance Scale, and handgrip strength was measured with a Jamar^®^ dynamometer.

**RESULTS:**

Patients with rheumatoid arthritis had significantly lower Berg Balance Scale scores (p<0.0001) and a markedly higher likelihood of experiencing falls than controls (OR 6.2; 95%CI 1.48–28.2). In univariate analysis, age (p<0.0001), handgrip strength (p=0.002), hypertension (p=0.02), tobacco exposure (p=0.0008), antimalarial use (p=0.02), and methotrexate use (p=0.007) were significantly associated with Berg Balance Scale scores. However, after multivariable adjustment, only antimalarial use (β=-2.75;95%CI -5.12 to -0.38; p=0.02), handgrip strength (β=0.09; 95%CI 0.01–0.17; p=0.01), and tobacco exposure (β=-1.90; 95%CI -3.20 to -0.62; p=0.004) remained independently associated with balance performance.

**CONCLUSION:**

Postural imbalance appears to be an important and potentially modifiable feature of rheumatoid arthritis. Routine balance assessment, together with interventions targeting muscle strength and smoking cessation, may help reduce fall risk and improve functional outcomes. Further studies are needed to clarify the relationship between antimalarial use and balance impairment.

## INTRODUCTION

Postural imbalance is a clinically relevant but still underexplored manifestation of rheumatoid arthritis (RA). Balance, a sophisticated neuromuscular function, refers to the body’s ability to control its center of gravity with minimal postural sway and maximum stability within the base of support^1^. Impaired postural control leads to increased postural sway, which, even in healthy individuals, is associated with a higher risk of falls^2^. In patients with RA, balance may be impaired by multiple mechanisms, including reduced joint proprioception secondary to foot deformities and lower-limb arthritis, muscle weakness, limited joint mobility, and, in some cases, central nervous system involvement^1^. Consequently, individuals with RA have a higher risk of falls than healthy individuals^1^. This elevated fall risk is further exacerbated by factors such as the use of antidepressants, impaired vision, and antihypertensive medications^1^. The concern surrounding falls in RA patients is amplified by their frequently compromised bone health and an increased propensity for fractures, largely attributable to reduced bone mass and the high prevalence of osteoporosis within this population^3^. In RA, bone loss is associated with the underlying inflammatory process, characterized by elevated levels of TNF-alpha, IL-6, and IL-1, which promote osteoclast activation and bone resorption. Moreover, the widespread use of glucocorticoids in RA management, although effective for controlling inflammation, is another significant risk factor for the development of secondary osteoporosis. Despite major advances in RA treatment, particularly the widespread use of disease-modifying antirheumatic drugs (DMARDs) and efforts to minimize glucocorticoid exposure, fracture risk remains substantial. A prospective observational study conducted in Japan demonstrated an increase in both vertebral and non-vertebral fractures among patients with RA between 2011 and 2023^4^.

A meta-analysis including 34 studies and 24,123 participants reported a pooled prevalence of 34% (95%CI 29–38%) for at least one fall and 16% (95%CI 12–20%) for recurrent falls among adults with RA^5^. In another study involving 4,996 individuals with RA, 10.1% reported experiencing at least one fall, 2.2% reported multiple falls, and 19.2% expressed a fear of falling within the previous 6 months^6^. Notably, those who reported falls were significantly more likely to have sustained fractures during the same period compared to non-fallers (14.7 versus 1.1%)^1^.

Given this background and the scarcity of data on postural balance among Brazilian patients with RA, we evaluated postural balance and fall risk in a cohort of individuals with RA. Additionally, we aimed to examine potential associations with age, disease duration, disease activity, muscle strength, and comorbid conditions. Generating local data is particularly important because genetic background, environmental factors, and disparities in access to healthcare may influence disease severity, functional outcomes, and fall risk in patients with RA.

## METHODS

This is a cross-sectional case–control study with a convenience sample of RA patients from a single Rheumatology Center that cares for patients from the Public Health Service in Brazil. Patients were recruited from March 2024 to April 2025. The project was approved by the Institutional Committee of Ethics in Research under protocol number 6.759.253, and included patients were invited to participate according to appointment order. To be included, the patients should fulfill at least 6 points in the American College of Rheumatology/European League Against Rheumatism (ACR/EULAR) Classification criteria for RA^7^, be over 18 years of age, and provide written informed consent. Individuals with neurological disorders, lower-limb orthopedic conditions, or amputations were excluded. Participants were consecutively recruited according to the order of their scheduled outpatient consultations, provided they met the predefined inclusion and exclusion criteria and expressed willingness to participate in the study. Companions accompanying the patients were invited to serve as controls and were matched to the study group by sex and age.

### Data collection included

Epidemiological and clinical data: age, sex, self-declared ethnic background, tobacco use, disease duration, presence of rheumatoid factor, current medications, comorbidities, and the number of falls in the previous year. Information was obtained through medical chart review and direct patient interviews.

Disease activity assessment: Disease activity was evaluated using the DAS28-ESR (Disease Activity Score using 28 joints and erythrocyte sedimentation rate), CDAI (Clinical Disease Activity Index), and SDAI (Simplified Disease Activity Index)^8^. The DAS28-ESR incorporates the number of tender and swollen joints (out of 28), the ESR value, and the patient’s general health status or global disease activity assessed via a 100 mm visual analog scale (VAS). The CDAI is based on the 28-joint tender and swollen counts, the patient’s global assessment of disease activity (0–10 scale), and the evaluator’s global assessment (0–10 scale). The SDAI includes the same parameters as the CDAI, with the addition of the C-reactive protein (CRP) level^8^.

Functional balance evaluation: The Berg Balance Scale (BBS) was a validated instrument used to assess patients’ static and dynamic balance across 14 tasks. Each item is scored on a scale from 0 to 4, yielding a maximum total score of 56 points^9^. Scores below 45 suggest increased risk of falling, with scores under 40 being associated with an almost certain risk of falls^10,11^.

Handgrip strength measurement: Handgrip strength was assessed using a Jamar^®^ dynamometer, with participants seated and elbows flexed at 90 degrees. In total, three measurements were taken, and the highest value was used for analysis. Measurements were performed on the dominant hand.

Statistical analysis: Data were organized in frequency and contingency tables. The Shapiro-Wilk test was applied to determine the distribution of continuous variables. Comparisons of BBS scores across clinical, epidemiological, and therapeutic subgroups were performed using the Mann-Whitney U test. Correlations between BBS and numerical variables were evaluated using Spearman’s rank correlation coefficient. A p-value of <0.05 was considered statistically significant. Variables that demonstrated an association with BBS at a significance level of p<0.1 were subsequently entered into a multivariate analysis to assess their independence. All statistical computations were carried out using MedCalc^®^ version 10.0.

## RESULTS

A total of 101 patients with RA and 94 healthy controls were included in the study. None were excluded. Table 1 summarizes the epidemiological characteristics of patients and controls, the clinical characteristics of the RA group, and the results of the BBS and handgrip strength assessments. Compared with controls, patients with RA had significantly lower BBS scores, reported more falls, and exhibited lower handgrip strength.

**Table 1:** Sample description and controls data pairing.

	Rheumatoid arthritis	Controls	p-value
Number (n)	101	94	
Female sex—n (%)	90 (89.1)	76 (80.8)	0.11
Age-years—median (IQR)	58 (52–65)	55 (47–5)	0.10
Tobacco exposure (*)	41 (40.5)	15 (15.9)	0.0001
BBS values—median (IQR)	55 (53–56)	56 (56–56)	0.0001
BBS<45 (n)	6 (5.9%)	0	0.02
At least one fall last year (n)	12 (11.8%)	2 (2.1%)	0.01
Hand grip strength values—median (IQR)	18.0 (12.5–21.0)	22.0 (18.0–30.0)	<0.0001
Positive rheumatoid factor—n (%)	70/93 (75.2)		
Disease duration—years—median (IQR)	12.0 (8.0–17.0)		
Treatment—n (%)			
Antimalarials	7 (6.9)		
Methotrexate	35 (34.6)		
Leflunomide	46 (45.5)		
Anti-TNF	19 (18.8)		
Anti-IL-6	4 (3.9)		
Rituximab	4 (3.9)		
iJAK	22 (21.7)		
Prednisone	30 (29.7)		
Inflammatory indexes—median (IQR)			
DAS 28 ESR	2.8 (2.22–3.58)		
CDAI	4.0 (0–10.0)		
SDAI	4.1 (0.74–10.7)		
ESR—mm	32.0 (21.0–45.0)		
CRP—mg/dL	12.0 (8.0–18.5)		
Comorbidities—n (%)			
Arterial hypertension	49 (48.5)		
Diabetes mellitus	10 (9.9)		

n: number; IQR: interquartile range; BBS: Berg Balance Scale; TNF: tumor necrosis factor; iJAK: Janus kinase inhibitors, ESR: erythrocyte sedimentation rate; CRP: C-reactive protein; DAS-28: disease activity score using 28 joints; CDAI: clinical disease activity index; SDAI: simplified disease activity index.

*Current and former smokers.

Table 2 presents the correlations between BBS scores and continuous variables. Age showed a moderate negative correlation with BBS scores, whereas handgrip strength demonstrated a modest positive correlation.

**Table 2:** Correlation studies of Berg Balance Scale with numerical variables.

Variable	R	95%CI	p-value
Hand grip values	0.29	0.10 to 0.47	0.002
Age	-0.41	-0.56 to -0.22	<0.0001
DAS 28 ESR	-0.06	-0.26 to 0.14	0.55
CDAI	-0.06	-0.26 to 0.14	0.53
SDAI	-0.09	-0.30 to 0.12	0.40
ESR	-0.12	-0.31 to 0.08	0.22
C-reactive protein	0.05	-0.14 to 0.25	0.57
Disease duration	-0.17	-0.36 to 0.02	0.07

DAS-28 ESR: disease activity score using 28 joints and ESR; ESR: erythrocyte sedimentation rate; CDAI: clinical disease activity index; SDAI: simplified disease activity index.

Table 3 presents the distribution of BBS scores according to categorical variables. Lower BBS scores were significantly associated with tobacco exposure, antimalarial use, methotrexate use, and hypertension.

**Table 3:** Study of the Berg Balance Scale according to nominal variables.

Variable	Median BBS values in patients with the variable	Median BBS values in patients without the variable	p-value
Female sex	55.0 (52.7–56.0)	56.0 (53.0–56.0)	0.10
Tobacco exposure	54.0 (52.0–56.0)	55.0 (54.0–56.0)	0.008
Rheumatoid factor	55.0 (52.7–56.0)	54.0 (51.5–55.0)	0.35
Medications used (*)			
Antimalarials	53.0 (47.0–54.0)	55.0 (53.0–56.0)	0.02
Methotrexate	56.0 (54.0–56.0)	54.0 (52.0–56.0)	0.0007
Leflunomide	55.0 (52.0–56.0)	55.0 (53.0–56.0)	0.90
Anti-TNF	55.0 (54.0–56.0)	55.0 (52.0–56.0)	0.48
Jak inhibitors	55.0 (52.7–56.0)	55.0 (53.0–56.0)	0.61
Prednisone	55.0 (52.7–56.0)	55.0 (53.0–56.0)	0.52
Antidepressants/anxiolytics	54.0 (52.0–56.0)	55.0 (53.0–56.0)	0.06
Comorbidities			
Arterial hypertension	54.0 (52.0–56.0)	56.0 (53.2–56.0)	0.02
Diabetes	54.0 (49.5–55.0)	55.0 (53.0–56.0)	0.13

(*) - anti-IL-6 and rituximab use not studied due to small sample. BBS: Berg Balance Scale; TNF: tumor necrosis factor.

Table 4 shows the results of the study variables’ independency. A model of multiple regression was built with BBS values as the dependent variable and entered all variables that are associated with BBS with p<0.10. Only antimalarial use, handgrip, and tobacco exposure remained as independent variables.

**Table 4:** Univariate and multivariable linear regression analyses of factors associated with Berg Balance Scale scores in patients with rheumatoid arthritis.

Variable	Univariate analysis β (95%CI)	p	Multivariable analysis β (95%CI)	p-value
Age	-0.086 (-0.149 to -0.022)	0.009	-0.023 (-0.090 to 0.045)	0.506
Handgrip strength	0.124 (0.045 to 0.202)	0.002	0.094 (0.018 to 0.171)	0.016
Disease duration	-0.075 (-0.140 to -0.011)	0.023	-0.046 (-0.110 to 0.018)	0.154
Antimalarial use	-2.512 (-5.008 to -0.016)	0.049	-2.754 (-5.121 to -0.387)	0.023
Methotrexate use	2.019 (0.721 to 3.316)	0.003	0.799 (-0.527 to 2.125)	0.235
Tobacco exposure	-1.748 (-3.018 to -0.478)	0.008	-1.916 (-3.208 to -0.624)	0.004

Dependent variable: Berg Balance Scale (BBS) score. β: regression coefficient; CI: confidence interval; CNS: central nervous system. Variables entered into the multivariable model were sex, age, disease duration, hypertension, handgrip strength, methotrexate use, antimalarial use, tobacco exposure, and use of antidepressants/anxiolytics. The multivariable model included all variables selected a priori based on clinical relevance and/or univariate analysis.

Table 4 presents the results of the multivariable regression analysis. A multiple linear regression model was constructed using the BBS score as the dependent variable, with all variables showing an association with the BBS score at p<0.10 entered into the model. After adjustment, only antimalarial use, handgrip strength, and tobacco exposure remained independently associated with BBS scores.

## DISCUSSION

This study found that Brazilian individuals with RA had poorer postural balance and experienced more falls than healthy controls. In this cohort, postural imbalance was independently associated with reduced handgrip strength—a surrogate marker of overall muscle function—antimalarial use, and tobacco exposure.

Handgrip strength is widely recognized as a simple and cost-effective tool for assessing global muscle strength, overall health status, and physical activity levels^11,12^. It has been extensively used as a predictor of sarcopenia^12^. Recent evidence emphasizes that weakness in the lower extremities constitutes a critical risk factor for falls, highlighting the importance of lower-limb muscle strength and quality in maintaining postural control and preventing imbalance. Notably, the primary hip abductors—the gluteus maximus, medius, and minimus—play an essential role in effective recovery of postural stability^13^.

According to Torii et al.^14^, sarcopenia affects approximately one-third of patients with RA, compared to 10% in the general population. Unlike primary sarcopenia, typically seen in the elderly, RA-related sarcopenia may occur in younger adults and is characterized by loss of skeletal muscle mass with preserved or increased fat mass^15^, differing from the generalized weight loss of cachexia or heart failure^16^. Its pathogenesis involves immune dysregulation, hormonal imbalance, and chronic cytokine-mediated inflammation (TNF-α, IL-1, IL-6, IFN-γ), which promote proteolysis and impaired muscle regeneration^17^. Despite therapeutic advances in inflammation control and joint protection, interventions targeting muscle recovery remain limited. No pharmacological therapy has been approved for sarcopenia; however, resistance training shows strong evidence for improving muscle strength and mass^18^.

In this study, antimalarial use was also associated with postural imbalance. Although rarely emphasized, ototoxicity is a recognized adverse effect of these drugs. Chloroquine-induced ototoxicity tends to appear acutely, whereas hydroxychloroquine-related effects emerge with prolonged use^19^. According to Muller et al., the inner ear’s melanocytes, crucial for cochleovestibular function, may undergo structural and functional damage, and melanin loss can lead to reactive oxygen species accumulation, which is toxic to cochlear hair cells^20^. Clinical consequences include sensorineural hearing loss, tinnitus, and imbalance^21^. The association observed in the present study between postural imbalance and antimalarial use requires confirmation in larger, independent cohorts. Furthermore, investigations that consider cumulative drug exposure may provide additional insights into this relationship. Unlike systemic lupus erythematosus, in which antimalarials are a cornerstone of therapy, RA can usually be managed with several alternative disease-modifying agents. Accordingly, this issue warrants further investigation and clarification.

Tobacco exposure remained independently associated with poorer balance performance in this cohort. In a study of 4,224 Japanese men, smokers performed significantly worse on one-leg balance tests than nonsmokers^22^. Similarly, forest workers who smoked showed greater balance impairment^23^. Nicotine has deleterious effects on motor, sensory, cognitive, and attentional functions, weakens postural muscles, reduces inner ear perfusion, and disrupts vestibular function^24^. Additionally, tobacco use worsens clinical outcomes in RA, potentially impairing lower-limb joint function—an essential component of postural control^25^. These combined effects suggest that smoking may influence both systemic and vestibular pathways implicated in postural control, potentially contributing to balance disturbances and offering a plausible explanation for the observed association.

In agreement with previous studies, RA patients in our study experienced significantly more falls than controls (OR 6.2). This finding raises clinical concern given that RA is frequently associated with reduced bone mass and increased fracture risk due to both disease-related bone loss and prolonged glucocorticoid use. Osteoporotic fractures, particularly hip fractures, are a major cause of morbidity and mortality worldwide^4^.

The present study has several limitations, including a relatively small sample size and the predominance of participants with low disease activity, which limited a more comprehensive assessment of the impact of systemic inflammation. In addition, fall history was based on self-reported data from the preceding year and may therefore be subject to recall bias. The absence of a detailed clinical evaluation of lower-limb joint status further restricted a deeper understanding of the mechanisms underlying postural imbalance in RA. Despite these constraints, the findings may offer clinically meaningful insights by indicating that antimalarial use, muscle weakness, and tobacco exposure could be associated with impaired balance in Brazilian patients with RA. Although exploratory, these findings highlight the importance of addressing potentially modifiable risk factors during routine clinical care. A multidisciplinary approach integrating optimal inflammatory control, structured resistance exercise, nutritional optimization, and smoking cessation may help reduce sarcopenia, improve postural balance, and ultimately enhance functional outcomes in patients with RA.

## CONCLUSION

These findings suggest that postural imbalance is a clinically relevant feature of RA that may involve factors beyond inflammatory disease activity alone. The independent associations with reduced muscle strength and tobacco exposure suggest that neuromuscular function and smoking-related factors may contribute substantially to impaired balance, highlighting opportunities for targeted rehabilitation and preventive interventions. The association observed with antimalarial use warrants further investigation to clarify whether it reflects a true treatment effect, disease-related characteristics, or residual confounding. Routine assessment of postural balance may help identify patients at increased risk of falls and support multidisciplinary interventions aimed at preserving mobility, reducing fall risk, and maintaining functional independence.

## Data Availability

The datasets generated and/or analyzed during the current study are available from the corresponding author upon reasonable request.
